# Lipidomics in Major Depressive Disorder

**DOI:** 10.3389/fpsyt.2018.00459

**Published:** 2018-10-15

**Authors:** Andreas Walther, Carlo Vittorio Cannistraci, Kai Simons, Claudio Durán, Mathias J. Gerl, Susanne Wehrli, Clemens Kirschbaum

**Affiliations:** ^1^Biological Psychology, TU Dresden, Dresden Germany; ^2^Biomedical Cybernetics Group, Biotechnology Center (BIOTEC), Center for Molecular and Cellular Bioengineering (CMCB), Center for Systems Biology Dresden (CSBD), Department of Physics, TU Dresden, Dresden, Germany; ^3^Brain Bio-Inspired Computing (BBC) Lab, IRCCS Centro Neurolesi “Bonino Pulejo”, Messina, Italy; ^4^Lipotype GmbH, Dresden, Germany

**Keywords:** major depressive disorder, depression, lipidomics, machine learning, computational psychiatry, cortisol, inflammation, prostaglandin

## Abstract

Omic sciences coupled with novel computational approaches such as machine intelligence offer completely new approaches to major depressive disorder (MDD) research. The complexity of MDD's pathophysiology is being integrated into studies examining MDD's biology within the omic fields. Lipidomics, as a late-comer among other omic fields, is increasingly being recognized in psychiatric research because it has allowed the investigation of global lipid perturbations in patients suffering from MDD and indicated a crucial role of specific patterns of lipid alterations in the development and progression of MDD. Combinatorial lipid-markers with high classification power are being developed in order to assist MDD diagnosis, while rodent models of depression reveal lipidome changes and thereby unveil novel treatment targets for depression. In this systematic review, we provide an overview of current breakthroughs and future trends in the field of lipidomics in MDD research and thereby paving the way for precision medicine in MDD.

## Introduction

Major depressive disorder (MDD) is a psychiatric illness with devastating consequences with regard to personal and social functioning as well as physical health (1, 2). According to the Diagnostic and Statistical Manual of Mental Disorders (DSM-5), MDD is a heterogeneous disorder, defined and diagnosed on the basis of its core symptoms of a depressed mood or anhedonia and a combination of four of nine other symptoms, such as changes in appetite, sleep, fatigue, concentration, feelings of worthlessness and suicidal ideations persisting for the majority of the day over at least a 2 week period (3). The World Health Organization has declared MDD the leading cause of disability with over 300 million people being affected worldwide (4). Regrettably, no sizeable improvement in population mental health for MDD has been achieved during the last decade (5). Diagnosing and treating MDD is complicated due to its heterogeneous illness presentation exemplified by anhedonia, considered a cardinal feature of MDD, but is truly present in only up to 50% of patients (6). The subjective nature of the patients' report of symptoms makes clinical judgment based on a person's history and cultural norms a difficult task. The high non-responder rates to standard antidepressant treatment present further challenges to clinical practice (7, 8). These factors contribute to low diagnostic reliability and the potential for misdiagnosis (9, 10).

Therefore, during the last few decades, tremendous efforts have been undertaken in order to identify a diagnostic biomarker for MDD, enabling a reliable diagnosis and potentially identifying treatment targets for novel therapeutic approaches. Although advancements in elucidating the pathophysiology of MDD have been achieved (11), one needs to acknowledge that the reductionist single biomarker approach employed during the last decades has been a failure (12). It has become increasingly clear that the pathophysiology of MDD depends on a wide array of biological parameters that cannot be measured by a single biomarker and that only a systems approach might be successful in identifying diagnostic bio-signatures for MDD.

Newly developed high-throughput screening technologies, which facilitate the quantification of omics data, offer new approaches for systems analyses with regard to MDD (13). New computational methods are currently revolutionizing biomarker research by using unsupervised data driven approaches such as machine intelligence or deep learning; these provide increased analytical power in order to come to grips with the complex pathophysiology of MDD (14–16). These computational approaches are capable of delivering multi-parametric disease signatures based on different omics data sets that can be used to identify functional networks (17).

Although studies using omics approaches (e.g., genomic, proteomic, brain connectomics, metabolomics) have increased exponentially during the last 18 years, achieving breakthroughs for example in the field of genome-wide or neurophysiological classification of MDD (18, 19), reproducibility has proved challenging the field. Lipidomics is no exception (12). However, this situation is now improving with the developments of new lipidomics platforms (20) that provide reliable absolute quantitation and inter-site reproducibility (21), which are the basis for clinical applications (12). In this systematic review we will provide an up-to-date overview of existing studies in lipidomics and MDD or rodent models of depression.

## The lipidome in MDD

The lipidome—the complete lipid profile of an organism—has central roles in most aspects of cell biology (22). The human organism invests substantial resources for the production of thousands of molecular species attributable to eight different lipid categories with multiple classes and sub-classes (23). These classes are strongly interconnected and perturbations in a lipid species often result in global lipidome changes (23, 24). An enormous variety of different functions is carried out by lipids, such as energy storage by triacylglycerol (TAG) in lipid droplets or membrane formation and trafficking by amphipathic lipids such as glycerophospholipids (e.g., phosphatidylcholines [PC], phosphatidylethanolamines [PE], and phosphatidylinositols [PI]) (25). Lipids also determine the localization and function of proteins in the cell membranes of neurons and may further act as neurotransmitters (26). Many lipid structures have been shown to be essential for neuronal signaling and survival (27–29), and thereby critically influence an individuals' mood and behavior (26). Lipids also function to sub-compartmentalize cell membranes, forming functional platforms (lipid rafts) that operate in signaling and many other membrane activities (30). Finally, lipids act as second messengers in signal transduction, also regulating glucocorticoid action as well as inflammatory processes (25). Therefore, there is a plethora of lipid markers potentially related to MDD. Reviews and meta-analyses investigating the action of single lipids in MDD suggest that polyunsaturated fatty acids (e.g., omega-3 and omega-6) (31), cholesterol (32), as well as lipids of the sphingomyelin-ceramide (SM-Cer) pathway could be crucially related to mood disorders (33, 34). However, as outlined above, lipids are strongly interconnected and hierarchically organized so that a systems approach based on networks will be an indispensable tool to reveal the role and mechanisms of the lipidome in MDD. Since lipid nomenclature is not yet standardized, in the following, we will report lipid species as in original reports (35).

**Figure 1:**
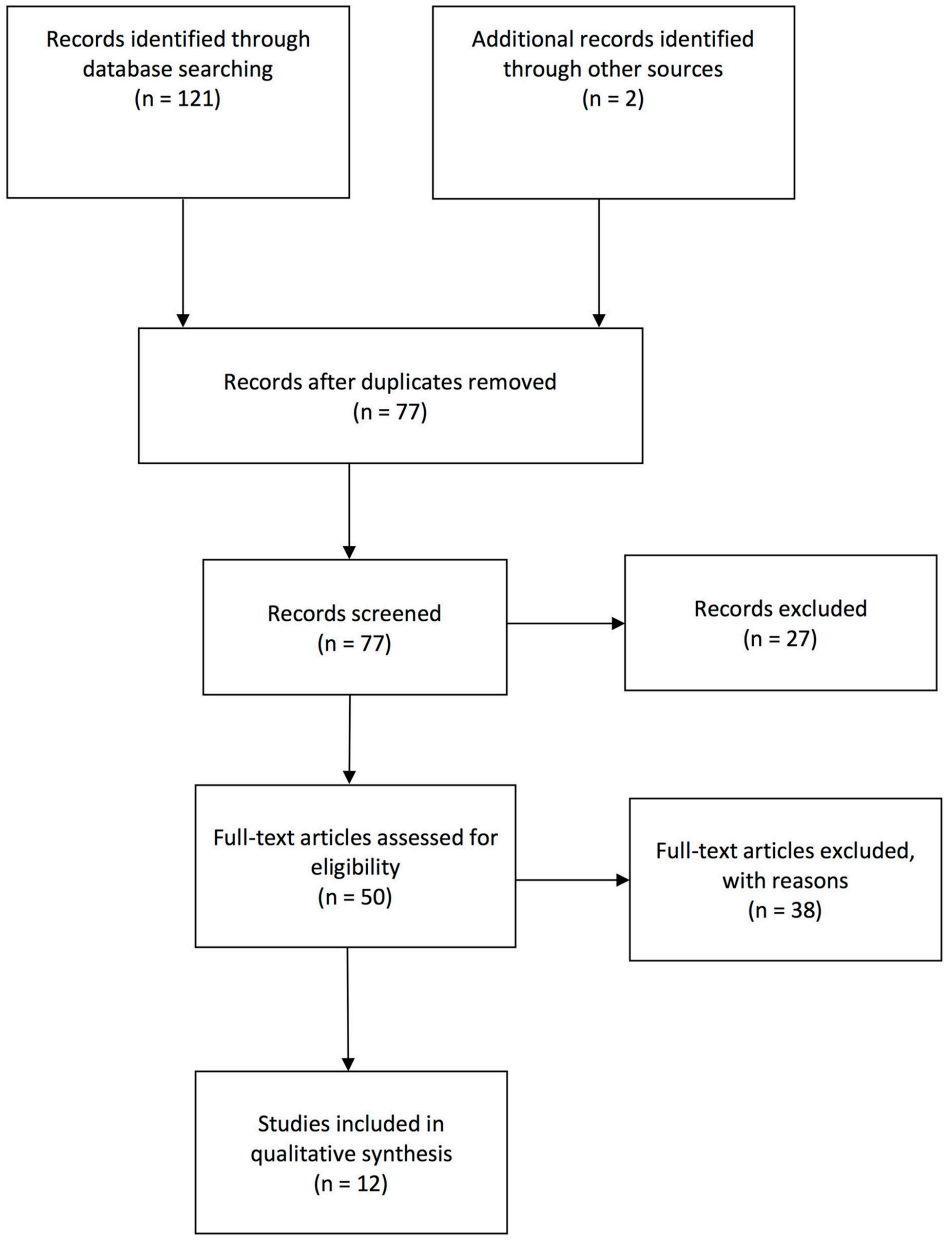
Flow chart visualization of systematic search process according to PRISMA guidelines.

**Figure 2:**
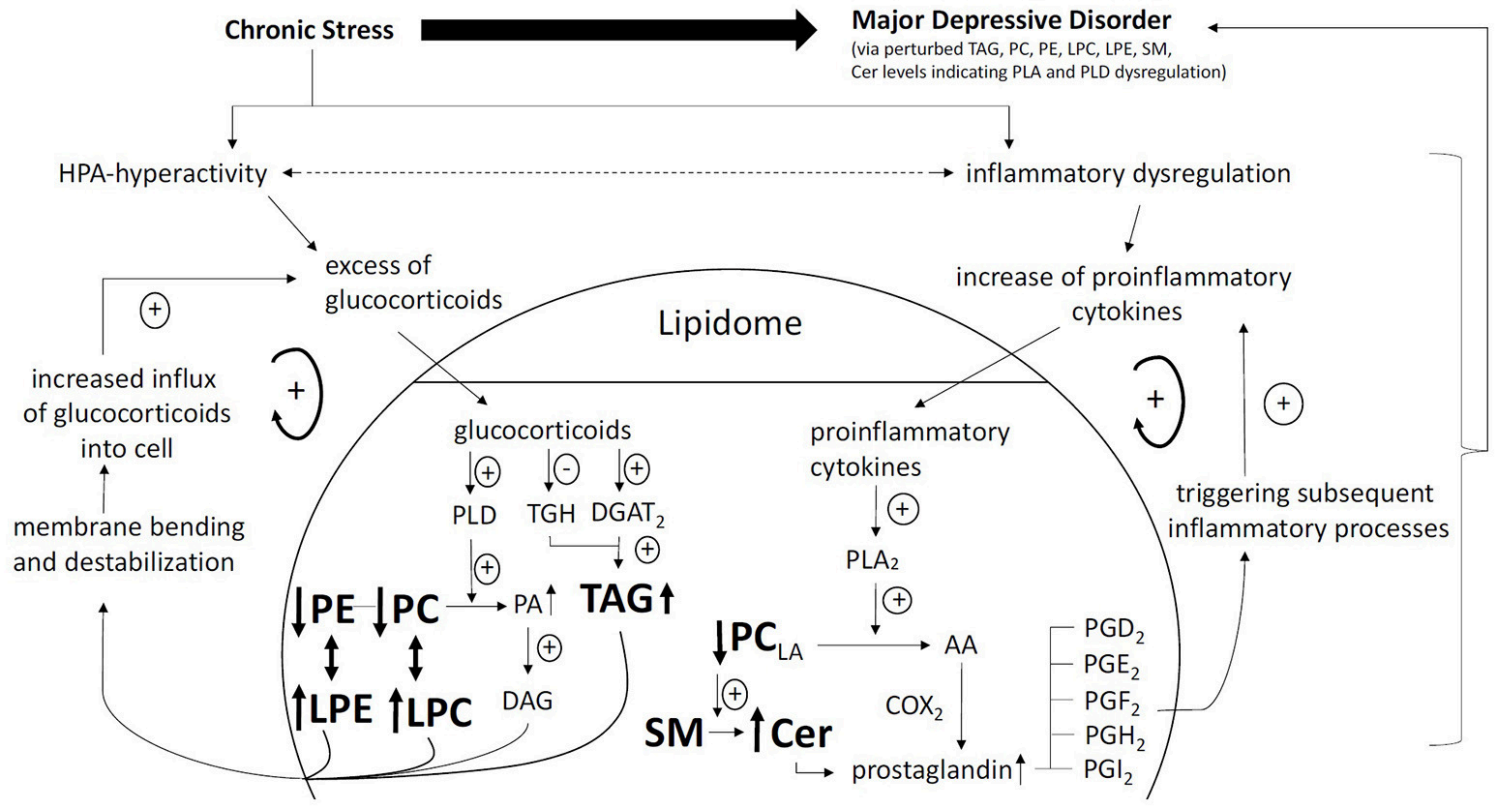
Pathway linking chronic stress, HPA hyperactivity, increased inflammation, lipidomic dysregulation, and development of MDD. *Glucocorticoid positive spiral pathway A:* Chronic stress leads to HPA hyperactivity. Increased levels of glucocorticoids increase PLD activity (48). Increased PLD activity leads to increased turnover of phosphatidylcholines (PC) and phosphatidylethanolamine (PE) to phosphatidic acid (PA) and also lysophosphatidylcholine (LPC) and lysophatidylethanolamine (LPE). Due to is chemical properties, PA is swiftly converted to diacylglycerol (DAG). DAG, LPC, and LPE cause membrane bending and destabilization allowing more influx of glucocorticoids into the cell. *Glucocorticoid positive spiral pathway B:* Increased levels of glucocorticoids decrease triacylglycerol hydrolase (TGH) expression. Increased levels of glucocorticoids increase triacylglycerol (TAG) biosynthesis via increase of (diacylglycerol acyltransferase 2) DGAT2. Reduced TGH expression and increased TAG biosynthesis lead to increased levels of TAG (49). TAG in turn is related to increased levels of glucocorticoids. *Inflammatory positive spiral pathway:* Chronic stress leads to inflammatory dysregulation. An excess of proinflammatory cytokines and phase reactants increase phospholipase A2 (PLA2) (50). Increased PLA2 activity leads to increased turnover of linoleic acid (LA) containing phosphatidylcholines (PC_LA_) to arachidonic acid (AA). AA is subsequently converted to prostaglandins (PG) of which the series 2 is proinflammatory (e.g., PGA2, PGD2, PGE2, PGF2, PGH2, PGI2). PG further increase inflammatory reactions (40). PC degradation is associated with SM-Cer turn over further increasing PG levels (22). *Lipid Nomenclature:* following annotations are used: Lipid class <sum of carbon atoms>: <sum of double bonds>; <sum of hydroxyl groups>, e.g., SM 34:1;2 signifies a SM species with 34 carbon atoms, 1 double bond and 2 hydroxyl groups in the ceramide backbone. Lipid molecular subspecies annotation (35) contains additional information about the exact identity of their fatty acids, the exact position of which cannot be discriminated in relation to the glycerol backbone (*sn*-1 or *sn*-2). This is indicated by a dash: “-.” For example, PC 18:1;0-16:0;0 denotes a phosphatidylcholine with a 18:1;0 (18 carbon atoms, 1 double bond, 0 hydroxylation) and a 16:0;0 fatty acid. CE 18:1;0 denotes a cholesteryl ester with a 18:1;0 fatty acid.

We will describe the systematic search process for studies applying lipidomic approaches in MDD or rodent models of depression (see Figure 1). Subsequently, we will review the identified studies with regard to their biological interpretation and discriminative power for MDD classification (see Table 1) and compare identified lipid species between studies (see Supplementary Table 1). Finally, we will integrate the findings into a model in which the relationship between the lipidome and MDD is mediated via dysregulated processes in the hypothalamus-pituitary-adrenal (HPA) axis and the immune system (see Figure 2).

**Table 1:** Summary of included studies on lipidomics in MDD or rodent models of depression.

**Reference**	**Population**	**Depression status categorization**	**Behavioral assessment**	**Sample**	**Quantification method**	**Study design**	**Finding**
**HUMAN STUDIES**							
Chan et al. (36)	Human, patients with coronary artery disease (CAD), North America	Dimensional depression scale (CES-D: equal/above vs. below cut off for depression = 16)	–	Patients with CAD and elevated depressive symptoms *N* = 37, mean age: 60.4, Control patients with CAD but not elevated depressive symptoms *N* = 49, mean age: 62.8 Total *N* = 86	Targeted gas chromatography	Discovery	Phospholipid bio-signature including 10 species discriminated between groups with AUC 84%. Findings indicate phospholipid-proinflammatory dependent pathway in patients suffering from CAD and MDD.
Demirkan et al. (37)	Human, general population, Europe	Dimensional depression scales (HADS-D, CES-D)	–	Erasmus Rucphen Family (ERF; genetically isolated population located in Netherlands), mean age discovery: 53.09, mean age validation: 48.13, discovery *N* = 742, validation *N* = 753, Total *N* = 1,495	Targeted electrospray ionization tandem mass spectrometry	Discovery / Validation	In the discovery set SM 23:1 and PC O 36:4 emerged as significantly correlated to depression measures. In a second, independent validation set the association for PC O 36:4 was replicated.
Hennebelle et al. (38)	Human, patients with seasonal affective disorders (SAD), North America	MDD diagnosis by structured clinical interview (DSM-V)	–	unmedicated participants euthymic at baseline, who met depression criteria in winter, mean age: 46.7, *N* = 9	Targeted liquid chromatography tandem mass-spectrometry	Discovery	No differential lipids were identified. In SAD patients, 4 increased oxylipins were identified in winter pointing toward inflammatory states in SAD patients. Potential association between winter depression and changes in cytochrome p450- and sEH-derived oxylipins were suggested.
Kim et al. (39)	Human, female MDD patients (current-MDD & remitted-MDD) vs. HCs, Korea	MDD diagnosis by Mini-International Neuropsychiatric Interview (MINI) according to DSM-IV criteria	–	Korean population, female MDD patients with a current major depressive episode: *N* = 25, MDD with current remission: *N* = 25, controls: N = 25, Total *N* = 75	Targeted liquid chromatography–mass spectrometry	Discovery	Lipid species of the LPA and TAG classes were differential between current, remitted, and non-depressed individuals. Discrimination power for different comparisons ranged between 60% (remitted vs. controls) and 76% (current vs. controls).
Knowles et al. (40)	Human, general population, North America	Continuous index of MDD based on Mini-International Neuropsychiatric Interview (MINI)	–	Participant data stems from randomly selected participants of the San Antonio Family Study (SAFS) mean age: 49.47, *N* = 567	Targeted liquid chromatography electrospray ionization-tandem mass spectrometry	Discovery	PC O and PC P species were associated to a genetic risk for MDD. PCO and PC P species containing AA exhibited the greatest degree of genetic overlap with MDD.
Kuwano et al. (41)	Human, general population, Japan	MDD diagnosis by structured clinical interview (DSM-IV TR)	–	Drug-naïve MDD patients, *N* = 15, healthy controls *N* = 19	Targeted liquid chromatography-mass spectrometry	Discovery	No differences between the plasma lipidome of MDD patients and HCs was identified. Depressive symptoms correlated with plasma tryptophan-kynurenine and lipid related metabolites.
Liu et al. (42)	Human, general population, China	MDD diagnosis according to DSM-IV	–	Discovery *N*: drug-naïve MDD = 60, HC = 59. Validation N: drug-naïve/drug-treated MDD = 75, HC = 52 Mean age discovery, HC: 43.98, mean age discovery MDD: 42.42, mean age validation HC: 33.67, mean age validation MDD: 36.04.	Non-targeted, ultra-performance liquid chromatography coupled with quadrupole time-of-flight mass spectrometry	Discovery / Validation	A diagnostic biomarker consisting of five differential lipids (LPE 20:4, PC 34:1, PI 40:4, SM 39:1, and TAG 44:2) was suggested with an AUC of 0.855 (moderately depressed vs. HC) and 0.931 (severely depressed vs. HC). Total levels of LPC, LPE, PC, PE, PI, DAG, TAG lipid classes were elevated in depressed individuals, while total PE O and several SM species were decreased
**Rodent studies**	**Population**	**Phenotype**	**Behavioral assessment**	**Sample**	**Quantification method**	**Study design**	**Finding**
Chen et al. (43)	Rodents	Chronic restraint stress for 6 weeks	Tail suspension test	Male Wistar rats, experimental *N* = 12, Allium macrostemon treated depression group = 11, control group = 12, total *N* = 35	Liquid chromatography/ion trap-time of flight mass spectrometry. Liquid chromatography/triple quadrupole mass spectrometry.	Experimental	Chronic restraint stress was associated with increased LPCs and reduced PCs
Faria et al. (44)	Rodents	Chronic unpredictable stress	Forced swimming test	Male, C57/BL6 mice with mice myocardium control (CTL), age: 9 weeks old, experimental *N* = 5 and Control *N* = 5, *N* = 3 for independent experiments	Targeted liquid chromatography–mass spectrometry, phosphorous assay, tissue samples of brain and myocardium	Experimental	An increases on brain lipid class level in the relative content of PC and PE levels and a decrease in the relative content of PI levels in the stressed mice could also be observed
Lee et al. (45)	Rodents	Injections of 10 mg/kg of the antidepressants maprotiline, fluoxetine and paroxetine for 4 weeks	-	male Balb/C mice, weighing 20–30 g each, aged 6–8 weeks, *N* = 16	Targeted, high-performance liquid chromatography/mass spectrometry, tissue samples of prefrontal cortex, hippocampus, striatum, cerebellum	Experimental	Decreased PC and increased LPC species due to maprotiline and paroxetine were observed in the prefrontal cortex indicating increased PLA2 activity due to treatment.
Lee et al. (46)	Rodents	Injection of the antidepressant maprotiline 10 mg/kg for 4 weeks or maprotiline plus bilateral prefrontal cortical injections of antisense oligonucleotide to iPLA2	Forced swimming test	Balb/C mice weighing 20–30 g each, aged 6–8 weeks, *N* = 20	Targeted, high-performance liquid chromatography (HPLC)/mass spectrometry, tissue samples of prefrontal cortex	Experimental	Decreased levels in PCs containing PUFAs and increased LPC levels were identified after maprotiline treatment indicating increased PLA2 activity due to treatment.
Oliveira et al. (34)	Rodents	Chronic unpredictable stress for 4 weeks or subcutaneous injections with 40 mg synthetic CORT 40 mg/kg for 4 weeks	Elevated plus maze	Adult male Wistar rats, aged 2 months, different conditions: Control *N* = 10, CUS Paradigm N = 10, CORT injections *N* = 10, Vehicle control group (sesame oil injections) *N* = 10,	Targeted, liquid chromatography mass spectrometry, tissue samples of prefrontal cortex, amygdala, hippocampus, cerebellum	Experimental	Due to the CUS paradigm relative levels of LPC, PA, PG, Cer species were increased, while relative levels of PC, PC E and PE species were decreased. After exogeneous CORT administration several brain lipid alterations were observed: decrease in PCs and PEs; increase in LPCs, Cers, PAs, PGs

## Search strategy and study selection

Candidate studies were identified via PubMed/Medline, EMBASE and PsycINFO using the following search strategy, whereby separate searches were performed for human and rodent studies respectively. The applied key terms for human studies were [(depressive mood OR depressed mood OR depression OR major depression) AND (lipidomics OR lipidome)], whereas for rodent studies the key terms [(depression OR depressive behavior OR depressive-like behavior OR depressed mood OR depressive mood OR antidepressant OR chronic unpredictable stress OR chronic unpredictable mild stress OR learned helplessness OR inescapable foot shock OR pain-induced depression OR social defeat) AND (rodents OR rats OR mice) AND (lipidomics OR lipidome)] were used for searching in the title, abstract and/or any other field registered in the database. The search was restricted to English language journal articles published between database inception and 25th April 2018. The final set of articles was cross-validated and further completed based on prior reviews. In sum, 123 records were extracted. The set was refined by removing (i) duplicate entries (*n* = 46). Titles and abstracts were then screened for relevance, removing (ii) reviews, meta-analyses, case studies, meeting abstracts, study protocols, practical guidelines and books (*n* = 27). In a next step, the full text of the remaining 50 articles was assessed for eligibility, subsequently excluding all studies that did not deal with (iii) lipidomics (*n* = 6) or (iv) MDD or behavioral mouse models of depression (*n* = 29). In a final step, studies (v) with a special focus on bio-technologies (*n* = 3) were eliminated. Figure 1 shows the sample development throughout the selection process, reaching the final set of 12 studies for review including 7 human and 5 rodent studies (see Table 1). The study selection and eligibility screening were conducted according to the PRISMA guidelines (47).

## Lipidomic studies in MDD and rodent models of depression

### Human studies

In humans, a pioneering study by Demirkan et al. using unsupervised lipidomic analysis of 148 plasma phospho- and sphingolipid species in a sample of 742 individuals identified significant associations with a psychometric depression measure (Center for Epidemiological Studies-Depression Scale: CES-D) for the ratio sphingomyelin (SM) 23:1 to SM 16:0 and the absolute amount of PC (alkyl subclass) O 36:4, as well as the ratio of PC O 36:4 to Cer 20:0 (37). However, subsequent analysis of an independent replication dataset (*N* = 753) only revealed absolute levels of PC O 36:4 to be robustly inversely related to depressive symptoms. Though being robustly associated to the CES-D in this study, this lipid structure alone did not provide sufficient discriminating power to adequately differentiate between individuals suffering from MDD and healthy controls. The authors of the study further highlighted that PC O 36:4 is a potential target of phospholipase A2 (PLA2), which converts PC O 36:4 to lysophosphatidylcholines (LPCs). When PC O 36:4 is hydrolyzed by PLA2, arachidonic acid (AA) is produced, which is subsequently rapidly converted into inflammatory mediators (prostaglandins), potentially leading to increased neuro-inflammation, which has consistently been related to MDD (51). AA itself, however, is known to directly modulate neural cell function via different processes (e.g., membrane fluidity and polarization) and thereby AA could negatively affect brain function and contribute directly to depressive symptomatology (37) [see also the study by Knowles et al. section Lipidome associations with glucocorticoids and inflammatory markers. for lipid-inflammation dependent pathways in MDD (40)].

Liu et al., based on prior work (52), conducted a non-targeted lipidomic approach comparing 60 patients with diagnosed MDD with 60 healthy controls (HCs) in a discovery set and a validation set of a similar size (42). The authors reported that several differential lipid species were significantly correlated with depression severity measured by the Hamilton Depression Scale (HAMD) (42). Total levels of the following lipid classes were elevated in depressed individuals LPC, lysophosphatidylethanolamine (LPE), PC, PE, phosphatidylinositol (PI), DAG, and TAG, while total PE O and several SM species were decreased in depressed individuals. The authors, however, did not elaborate on the molecular mechanisms linking these lipid classes with MDD. Yet, negative associations between specific SM species and depressive symptoms were also reported by Demirkan et al. (37). An experimental study further investigated the effects of acid sphingomyelinase (Asm), which releases ceramides (Cer) from SMs (33). In this study, it was shown that therapeutic concentrations of two different antidepressants reduced Asm activity and Cer levels, while increased neuroplasticity and non-depressive behavior was observed (33). This suggests that SM levels and the turnover to Cer are dysregulated in MDD and restored with antidepressant medication. Furthermore, using unsupervised statistical approaches, Liu et al. extracted a combinatorial lipid marker including five lipids as diagnostic biomarker with an area under the curve (AUC) between 0.855 and 0.931 suggesting a good discriminative power for MDD and HCs. However, the identified five lipids LPE 20:4, PC 34:1, PI 40:4, SM 39:1, and TAG 44:2 were not further validated in subsequent lipidomic studies on MDD. Unfortunately, the authors did not provide values for the area under the precision/recall curve (AUPR), although unbalanced analysis was performed when comparing HCs (*N* = 111) to either moderately depressed (*N* = 78) or severely depressed individuals (*N* = 57). Here, it is important to note that AUC alone is not a good measure of performance in unbalanced data sets as represented by the comparison of moderately or severely depressed vs. HCs. Thus, combined reporting of AUC and AUPR is required. AUC alone can present an overly optimistic view of the algorithm's performance when there is a skew in the class distribution (53). Comparing these results, a metabolomics/lipidomic study reports an accuracy for their extracted combinatorial biomarker of 72.2% for the discrimination of MDD and HCs, which is significantly lower (54). The authors subsequently sub-classified MDD individuals into anxious depressed (*N* = 58) and melancholic depressed (*N* = 21) and compared these groups to HC (*N* = 97). Using this approach, an accuracy of 83.8% for melancholic depressed individuals (72% for anxious depressed individuals) was achieved. However, the authors neither reported AUC nor AUPR levels (54). This might therefore still overestimate the actual discriminative power since the false positive rate was not considered within the accuracy measure. In this study, of the lipids, PC and SM levels were decreased and Cer levels were increased, again indicating increased SM to Cer turnover by Asm hyperactivity (54).

A study by Kim et al. including 25 currently affected MDD cases, 25 remitted MDD cases, and 25 HCs, identified a lipid marker consisting of lysophosphatidic acid (LPA) 16:1, TAG 44:0, and TAG 54:8, discriminating between HCs and MDD cases with 76% accuracy (HCs vs remitted with 60% accuracy; LPA16:1, TAG52:6, TAG54:8, TAG58:10) (39). With regard to differentially elevated lipids in the three groups, an inconsistent picture emerged with no differential PC and PE species, while TAG and DAG species showed the most differentiating power and were significantly increased in MDD patients compared to controls (39). TAGs are the most abundant lipid species in the human organism and constitute a major source of energy, while lipoprotein lipase (LPL) enzymes such as adipose triglyceride lipase (ATGL) and hormone sensitive lipase (HSL) are responsible for the breakdown of TAG into DAG and free fatty acids (FFA) (55). In the brain, TAG degradation by LPL informs neurons and astrocytes about energy expenditure (56), while a dysfunction of these enzymes leading to increased TAG levels may underlie the increased fatigue observed in depressive disorders.

Chan et al. further investigated the lipidome in patients with coronary artery disease (CAD) and identified 10 phospholipids, which showed good discriminative power (84%) to differentiate between CAD patients with substantial depressive burden (CES-D ≥ 16) and those without (36). The identified phospholipids contained several proinflammatory compounds (e.g., linoleic acid [LA] or AA), which are released by PLA2 and are crucially implicated in proinflammatory processes (57, 58). This finding further underlines a lipid-inflammation-dependent pathway in MDD. In line with this, Hennebelle et al. report that in seasonal affective disorders (SAD), four oxylipins, which are oxidation products of polyunsaturated fatty acids, were increased in winter (38). Since oxylipins are involved in regulating proinflammatory processes, this suggests that season-dependent changes in omega-3/-6 metabolism underlie inflammatory states in SAD. However, it is important to mention that a smaller study by Kuwano et al. failed to identify any significant differences in the plasma lipidome of first-episode drug-naïve MDD patients (*N* = 15) compared to HCs (*N* = 19), but identifed two metabolites (tryptophan and kynurenine) that were significantly reduced in patients (41).

Therefore, due to the plethora of potential lipid species and inconsistent results of lipidomic studies in MDD, uncertainty prevails about which lipids are implicated in MDD pathophysiology and which lipids best segregate between MDD and HCs. Furthermore, no data on the time course of these processes exist in humans, while rodent studies provide some insight into the causal relation between the lipidome and depression models.

### Rodent studies

Important insights emerged from recent rodent studies investigating the lipidome in models of depression. One of the first rodent studies examining the lipidome in rodents used a 4 week antidepressant treatment and compared the prefrontal cortex of the rats with regard to changes in the lipidome (45). In the prefrontal cortex, robust significant reduction in the relative abundance of PC species (PC36:1, PC38:3, PC40:2, PC40:6, PC40:5, PC42:7, PC42:6, and PC42:5) and increases of LPC species (LPC16:0, LPC18:0, and LPC18:2) were identified in response to maprotiline and paroxetine, but not fluoxetine treatment indicating an increase in PLA2 activity and possible release of long-chain fatty acids on the rat brain lipidome (45). For maprotiline and paroxetine, an increase in Cer levels (Cer18:1/18:0, Cer18:1/20:0, Cer18:1/22:0,) was also observed, as well as a decrease in PE levels (PE36:4, PE36:5) and mixed changes with regard to the direction of change in SM levels (increase: SM18/16:0; decrease: SM18/24:0, SM18/24:1). PCs are the major lipids distributed in the cell membrane, while LPCs directly exert effects on membrane permeability, and SMs and Cers are also critically integrated in the membrane formation process. These results indicate that antidepressant treatments influence membrane formation, structure and permeability. Here, it is important to mention that the authors did not experimentally induce depression in the rodents and then treat them with antidepressant medication, but instead directly treated the rodents with antidepressant medication. Although this might be beneficial on a behavioral level, altering an organism's homeostasis might be related to adverse consequences with regard to its lipidome. However, the same group added behavioral testing to the reported lipidome changes for maprotiline and showed increased non-depressive behavior for maprotiline treatment (46). Interestingly, when rodents concomitantly experienced PLA2 suppression, increased depressive behavior could be observed indicating a crucial role of PLA2 activity with regard to antidepressant treatment (46). PLA2 hydrolyses the fatty acid from the sn-2 position of PCs, rendering the cleavage products available for subsequent biological actions increasing LPC, DAG, and TAG levels (59). Contrasting these results in a study using chronic restraint stress, the depressed rodents exhibited increased plasma levels of LPC (LPC18:1, LPC20:1, LPC-O16:2, LPC-O18:3) and decreased levels of PC (PC32:1, PC36:4, PC37:4, PC38:4, PC40:6, PC-O36:4, PC-O38:5) compared to control animals (43). However, a rodent study of chronic unpredictable stress (CUS; a commonly used paradigm in preclinical depression research) showed significant increases on brain lipid class level in the relative content of PC and PE levels in the stressed mice. A significant decrease in the relative content of PI levels could also be observed indicating membrane structure and function change due to chronic stress (44). Another study which used the CUS model revealed somewhat opposing changes in the rat brain lipidome and mainly in the prefrontal cortex, with increased LPC, LPE, and Cer levels and decreased PE, ether phosphatidylcholines (PC E), and SM levels (34). Interestingly, with regard to serotonin deficiency investigated in a genetic knockout model TPH2-/-, serotonin-deficient mice expressed corresponding differences in the lipid profile, with reduced levels in several PC, PE, PI species and increased levels in several LPC, LPE, and Cer species (60). Taken together, the rodent studies, unlike the human studies, form a more consistent picture, showing that experimentally induced depressive states reduced PC, PE, PI, and increased LPC, LPE, Cer, DAG and TAG levels.

## Lipidome associations with glucocorticoids and inflammatory markers

After more than 30 years of biomarker research in MDD, hypercortisolism and chronic low grade inflammation are considered to be the two most consistent findings and the most salient biomarkers in MDD research (11). In the following, we provide an overview of the functional cross-talk between glucocorticoid secretion, inflammatory processes and the lipidome.

Studies by Mocking and colleagues have shown that in patients with recurrent MDD, salivary cortisol was negatively associated to fatty acid-metabolism in three fatty acids (arachidonic acid (AA), eicosapentaenoic acid (EPA), docosahexaenoic acid (DHA) (61). Furthermore, in a longitudinal study including 70 MDD patients and 51 matched controls, fatty acid-metabolism showed a significantly stronger negative relationship to salivary cortisol in patients than in the controls, while a stronger negative relationship between fatty acid-metabolism and cortisol levels was predictive for non-response to antidepressants in patients (62). Importantly, a rodent study examined the lipidome after exogeneous corticosterone administration and several brain lipid alterations were observed [decrease in: PCs, PE; increase in: LPC, Cer, PA, phosphatidylglycerol (PG)], revealing a tight interconnectedness between glucocorticoids and brain lipids (34). However, other studies reported contradictory results with regard to chronic stress (44), or found that there was no effect of corticosterone administration on Cer levels or acid sphingomyelinase (Asm), which metabolizes SM to Cer (33). However, in these contradictory studies, differing forms of application and dosages were used (oral 0.25 mg mL^−1^ via drinking water vs. subcutaneous injections of 40 mg kg^−1^) suggesting that higher doses of corticosterone administration reveal stronger changes in the rat brain lipidome. *In vitro* examinations further showed that dexamethasone-treated M-1 cells (a mammalian cell line from the cortical collecting duct) express higher phospholipase D (PLD) activity, which hydrolyzes PC to PA, subsequently leading to vesicle formation, budding, and fission from neurons (48). Increased PLD activity has also been related to neuro-inflammatory states via astrogliosis (63).

In line with this, a recently conducted lipidomic and genetic analysis in patients with MDD identified a shared genetic etiology between MDD and PC indicating markers of the PC-inflammation pathway as diagnostic markers for MDD (40). In addition, MDD patients were shown to demonstrate higher levels of AA and C-reactive protein (CRP; a marker for systemic inflammation) than those in HCs. In patients, AA correlates positively with CRP levels (64). Pro-inflammatory markers such as CRP have been shown to feature upstream in the inflammatory process, triggering PCs containing AA, which is subsequently released from the cell membrane via PLA2. This leaves necessary fatty acids available for prostaglandin production and further triggers subsequent pro-inflammatory processes (65). Pro-inflammatory markers were shown to increase PLA2 activity up to 14-fold (50). Recently, it has been shown that linoleic acid administration causes an increase in CRP secretion providing further evidence for a lipid-inflammation dependent pathway for MDD (57).

Therefore, it is suggested that lipidomic risk-profiles seem to predispose individuals to MDD and that these at-risk individuals subsequently become ill and show intensified glucocorticoid and inflammatory dysregulations. Thus, a testable model of the pathophysiology of MDD, as presented in Figure 2, emerges, suggesting that dysregulated candidate lipid networks increase the risk of MDD via direct effects on neurosignaling and their causal influence on hyper-cortisolism and systemic low grade inflammation (64).

## Conclusion

Due to the clear integration of multiple identified lipids in the pathophysiology of MDD based on our systematic review, lipidomics emerges as a powerful approach to identify a diagnostic biomarker for MDD, with promising results stemming from pioneering studies. However, prediction power is currently insufficient to extract clinically applicable lipid biomarkers for MDD. Furthermore, several differential lipid species were identified for MDD, although somewhat inconsistent between studies (see Supplementary Table 2). However, the emerging pattern of reduced PC, PE, PI, and increased LPC, LPE, Cer, TAG, and DAG levels in response to depressed states needs to be replicated in independent studies using lipidomics analysis (20). Recent advances in the field of computational psychiatry may further increase the predictive power of lipidomics for MDD (66), while quantitative analyses of identified lipid species and sub-species are needed to evaluate the most predictive lipids. However, potential pathways linking the lipidome to MDD highlight the inflammatory system as well as the glucocorticoid system as important mediators (see Figure 2). Furthermore, the one problem that all of the presented human lipidomic studies in MDD have in common is their cross-sectional nature. Not a single longitudinal lipidomic study in patients with MDD has been carried out so far. Thus, no data on possible alterations of lipid profiles with regard to changes of the disease state are available to date. Although potential molecular pathways linking MDD with lipid profiles via inflammatory and steroidal dysregulations have been suggested (34, 44), only longitudinal lipidomic studies in large human samples including classical inflammatory markers as well as glucocorticoids will provide clarification as to whether the proposed pathophysiologic model can be validated o r not. Finally, lipid pathway enrichment analyses of the identified lipids such as provided by LIPEA (67) will further shed light on the underlying mechanisms of how perturbed lipid pathways are contributing to MDD paving the way to precision medicine and thus providing novel drug targets for more effective treatments in MDD.

## Author contributions

AW designed and supported the systematic review and wrote the first draft of the manuscript. CC, KS, MG, CD, and CK contributed with important intellectual content and edited subsequent versions of the manuscript. SW supported the systematic review and created the art work.

### Conflict of interest statement

KS is shareholder and CEO of Lipotype GmbH. MG is employees of Lipotype GmbH. The remaining authors declare that the research was conducted in the absence of any commercial or financial relationships that could be construed as a potential conflict of interest.
