# Supplementary material for: Lipidomics in Major Depressive Disorder
Source: Front Psychiatry. 2018 Oct 15;9:459. doi: 10.3389/fpsyt.2018.00459 (PMC6196281; doi:10.3389/fpsyt.2018.00459)
Supplement: Supplementary file 1 [file Table_1.PDF]

Lipidomics in major depressive disorder: a systematic review

Supplementary Material

Walther, A.[1], Cannistraci, C.[2], Simons, K.[3] , Durán, C.[2], Gerl, MJ.[3] , Wehrli, S.[1] & Kirschbaum, C.[1]

[1] *Biological Psychology, TU Dresden, Dresden, Germany*
[2] *Biomedical Cybernetics, Biotechnological Center TU Dresden, TU Dresden, Germany*
[3] *Lipotype,* Biotechnological Center TU Dresden, *TU Dresden, Dresden, Germany*

Supplementary table 1. Overview of most differential lipids between studies

	Chan et al., 2018	Chen et al., 2014	Demirkan et al., 2013	Faria et al., 2014	Hennebelle et al., 2017	Kim et al., 2018	Knowles et al., 2017	Kuwano et al., 2018	Lee et al., 2009	Lee et al., 2012	Liu et al., 2016	Oliveira et al., 2016
**Lipid class**												
	LPC	LPC	Cer	CL		CE	PC O		Cer	LPC	LPC	APS
	PI	LPC O	LPC	PC		DAG	PC P		LPC	PC	LPE	CE
	PS	PC	PC	PE		LPA			PC	PE	PC	Cer
	SM	PC O	PC O	PI		LPC			PE		PC O	dhSM
		TAG	PE			LPI			SM		PE	LacCer
			PLPE			PI					PE O	LPC
			SM			PS					PI	LPE
						TAG					SM	PC
											TAG	PC E
												PE
												PI
												SM
												Sulf(2OH)
												TAG

Lipid species	Chan et al., 2018	Chen et al., 2014	Demirkan et al., 2013	Faria et al., 2014	Hennebelle et al., 2017	Kim et al., 2018	Knowles et al., 2017	Kuwano et al., 2018	Lee et al., 2009	Lee et al., 2012	Liu et al., 2016	Oliveira et al., 2016
		LPC 18:1	Cer 18:0			CE 20:5	PC P 36:5		Cer 18:1/18:0	LPC 16:0	LPC 16:0	Cer 16:0
		LPC 18:2	Cer 20:0			DAG 32:0	PC P 36:2		Cer 18:1/20:0	LPC 18:1	LPC 16:1	Cer 16:1
		LPC 20:1	LPC 20:5			DAG 36:1	PC P 38:5		Cer 18:1/22:0	LPC 20:4	LPC 18:1	Cer 18:1
		LPC O 16:2	PC 38:6			DAG 36:8	PC P 40:5		Cer 18:1/24:0	PC 32:0	LPE 16:0	Cer 22:1
		LPC O 18:3	PC O 34:2			LPA 16:1	PC O 36:2		LPC 16:0	PC 36:4	LPE 18:0	Cer 26:1
		PC 32:1	PC O 36:5			LPA 22:4	PC O 36:4		LPC 18:0	PC 38:3	LPE 18:1	dhSM 16:0
		PC 36:4	PC O 36:4			LPC 16:1	PC O 36:3		LPC 18:2	PC 38:4	LPE 18:2	dhSM 16:1
		PC 36:5	PC O 38:1			LPI 16:0	PC O 32:0		PC 32:0	PC 38:5	LPE 20:4	dhSM 18:0
		PC 37:4	PC O 38:4			LPI 18:2	PC O 32:1		PC 36:1	PC 38:6	PC 32:0	dhSM 18:1
		PC 38:4	PC O 38:5			PI 32:1	PC O 32:2		PC 38:3	PC 40:2	PC 34:1	dhSM 20:0
		PC 38:5	PC O 40:4			PI 32:2	PC O 34:0		PC 40:2	PC 40:4	PC 36:1	dhSM 22:0
		PC 38:6	PC O 40:6			PI 34:1	PC O 34:2		PC 40:5	PC 40:5	PC 38:2-1	dhSM 22:1
		PC 40:6	PE 32:2			PI 34:2	PC O 34:1		PC 40:6	PC 40:6	PC 38:5-1	dhSM 24:0
		PC O 36:4	PE 34:2			PI 34:3	PC O 36:0		PC 42:5	PE 34:1	PC 40:5	dhSM 24:1
		PC O 38:5	PE 36:5			PI 36:1	PC O 35:4		PC 42:6	PE 38:4	PC 42:1	dhSM 26:0
		TAG 58:12	PE 36:2			PI 36:2	PC O 34:4		PC 42:7	PE 38:5	PC O 34:2	dhSM 26:1
		TAG 60:12	PE 38:1			PI 36:3	PC O 38:5		PE 36:4	PE 38:6	PC O 42:5	dhSM 26:2
		TAG 62:13	PE 40:3			PS 34:2	PC O 38:4		PE 36:5	PE 40:4	PC O 44:5	LacCer 18:0
		TAG 62:14	PE 40:4			TAG 44:0	PC P 32:0		SM 34:0 (18:0/16:0)	PE 40:5	PE 36:1	LacCer 24:0
			PE 40:5			TAG 44:2	PC P 34:1		SM 42:0 (18:0/24:0)	PE 40:6	PE 36:3	LacCer 26:1
			PE 40:6			TAG 46:0	PC P 32:1		SM 42:1 (18:0/24:1)	PE 40:7	PE 36:4	LPC 16:0
			PE O 38:7			TAG 46:1	PC O 30:0				PE O 34:3	PA 40:5
			PE O 40:3			TAG 46:3	PC O 36:1				PE O 36:3	PA 40:6
			PLPE 18:1/18:1			TAG 48:1	PC O 36:5				PE O 36:5	PG 36:1
			PLPE 18:1/18:2			TAG 48:2	PC O 40:7				PE O 38:5	PC 36:1

PLPE 16:0/18:1	TAG 48:4	PC P 34:2	PE O 38:6	PC 38:0
PLPE 18:0	TAG 50:1		PE O 38:7	PC 38:1
SM 15:0	TAG 50:2		PE O 40:7	PC 38:2
SM 16:0	TAG 50:3		PI 34:1	PC E 34:1
SM 23:1	TAG 50:4		PI 36:1	PC E 36:1
SM 23:0	TAG 52:6		PI 38:4	PC E 38:2
	TAG 54:4		PI 38:6	PC E 38:3
	TAG 54:5		PI 40:4	PC E 38:4
	TAG 54:6		PI 40:5	PE 34:0
	TAG 54:7		PI 40:6	PE 34:1
	TAG 54:8		SM 36:1;2	PE 34:2
			SM 36:2;2	PE 36:0
			SM 38:1;2	PE 36:1
			SM 38:2;2	PE 36:2
			SM 39:1;2	PE 36:3
			SM 40:3;2	PE 36:4
			SM 41:1;2	PE 38:0
			SM 42:3;2	PE 38:1
			TAG 42:0	PE 38:2
			TAG 42:1	PE 38:3
			TAG 44:0	PE 38:4
			TAG 44:1	PE 38:5
			TAG 44:2	PE 38:6
			TAG 46:1	PE 40:4
			TAG 46:2	PE 40:5
			TAG 46:3	PE 40:6
			TAG 48:4	PE 42:5
			TAG 50:0	PE 42:6
				SM 16:0

SM 20:0
SM 22:0
SM 24:0
SM 26:0

Legend: APS: acylphosphatidylserine, BMP: bismonoacylglycerolphosphate, Cer: ceramide, IcCer: long-chain ceramide, LacCer: lactosylceramide, CE: cholesteryl ester, CL: cardiolipin, DAG: diacylglycerol, GM3: monosialodihexosylganglioside, Glu: glucosylceramide, HexCer: hexosylceramide, LPA: lysophosphatidic acid, LPC: lysophosphatidylcholine, LPC E: ether lysophosphatidylcholine, LPE: lysophosphatidylethanolamine, LPI: lysophosphatdylinositol, LPC: lysophosphatidylcholines, PA: phosphatidic acid, PC: phosphatidylcholine, PC E: ether phosphatidylcholine, PC O: alkylphosphatidylcholine, PE: phosphatidylethanolamine, PE O: alkenylphosphatidylethanolamine, PG: phosphatidylglycerol, PI: palmitic acid, PLPE: phosphatidylethanolamine-based plasmalogen, PS: phosphatidylserine, SM: sphingomyelin, dhSM: dihydrosphingomyelin, Sulf(2OH): 2- hydroxy N-acyl sulfatide, TAG: triacylglycerol, 24-OHC: 24(S)-hydroxycholesterol. For Chan et al. 2018 the differential lipids were named after their head-group and the relevant compounds within this structure. If lipid species could not be clearly assigned, the annotation of the primary report was retained.
